# The Ndst Gene Family in Zebrafish: Role of Ndst1b in Pharyngeal Arch Formation

**DOI:** 10.1371/journal.pone.0119040

**Published:** 2015-03-13

**Authors:** Beata Filipek-Górniok, Pernilla Carlsson, Tatjana Haitina, Judith Habicher, Johan Ledin, Lena Kjellén

**Affiliations:** 1 Dept. of Medical Biochemistry and Microbiology, Science for Life Laboratory, Uppsala University, Husargatan 3, PO Box 582, SE-751 23, Uppsala, Sweden; 2 Dept. of Organismal Biology, Science for Life Laboratory, Uppsala University, Norbyvägen 18A, SE-752 36, Uppsala, Sweden; Columbia University, UNITED STATES

## Abstract

Heparan sulfate (HS) proteoglycans are ubiquitous components of the extracellular matrix and plasma membrane of metazoans. The sulfation pattern of the HS glycosaminoglycan chain is characteristic for each tissue and changes during development. The glucosaminyl *N*-deacetylase/*N*-sulfotransferase (NDST) enzymes catalyze *N*-deacetylation and *N*-sulfation during HS biosynthesis and have a key role in designing the sulfation pattern. We here report on the presence of five NDST genes in zebrafish. Zebrafish *ndst1a*, *ndst1b*, *ndst2a* and *ndst2b* represent duplicated mammalian orthologues of *NDST1* and *NDST2* that arose through teleost specific genome duplication. Interestingly, the single zebrafish orthologue *ndst3*, is equally similar to tetrapod *Ndst3* and *Ndst4*. It is likely that a local duplication in the common ancestor of lobe-finned fish and tetrapods gave rise to these two genes. All zebrafish Ndst genes showed distinct but partially overlapping expression patterns during embryonic development. Morpholino knockdown of *ndst1b* resulted in delayed development, craniofacial cartilage abnormalities, shortened body and pectoral fin length, resembling some of the features of the *Ndst1* mouse knockout.

## Introduction

Heparan sulfate (HS) proteoglycans are found abundantly in basement membranes and on cell surfaces, where they function as co-receptors, store growth factors and participate in the generation of morphogen gradients [1]. The HS chains, covalently attached to different proteoglycan core proteins, display varying sulfation patterns in different cells and tissues [2–5], with very little variation between individuals [2]. This implicates that HS biosynthesis is a highly regulated process, and reflects the requirement for specific HS sulfation patterns in different biological processes.

HS is synthesized by a complex machinery of enzymes [6] and begins with the assembly of a “linkage tetrasaccharide”, consisting of glucuronic acid-galactose-galactose-xylose (GlcAβ1–3Galβ1–3Galβ1–4Xylβ1-), attached to a serine residue of the core protein. Polymerization takes place by addition of alternating *N*-acetylglucosamine (GlcNAc) and GlcA residues, a process catalyzed by the EXT (exostosin) polymerases. During polymerization, the HS chain undergoes a number of modifications. *N*-Deacetylase/*N*-sulfotransferase (NDST) is a bifunctional enzyme, which removes acetyl groups from GlcNAc residues and replaces them with sulfate groups. Mammalian genomes contain four different NDST genes (*NDST* 1–4). The *N*-deacetylation/*N*-sulfation reactions occur in a processive manner, creating stretches of *N*-sulfated regions and nonmodified *N*-acetylated domains interspersed with short mixed regions of alternating GlcNS and GlcNAc residues [7].

Other HS modification enzymes work mainly in *N*-sulfated domains resulting in highly sulfated NS-domains. These modifications include epimerization of GlcA into iduronic acid (IdoA), 2-*O*-sulfation of IdoA and more rarely GlcA residues, followed by 6-*O*- and 3-*O*-sulfation of glucosamine residues. In mammals, single isoforms catalyze epimerization and 2-*O*-sulfation, while three 6-*O*-sulfotransferases (6-OST1–3), including a splice variant (6-OST2S), and seven 3-*O*-sulfotranferases (3-OST1–7) have been characterized [6, 8]. Zebrafish express an even higher number of heparan sulfate biosynthetic enzymes. To this date, eight 3-OSTs [9] and four 6-OSTs [10–12] have been characterized in zebrafish. One 2-*O*-sulfotransferase and two zebrafish C5-epimerase genes, Glce-A and Glce-B, have also been reported [13, 14]. Despite the key role that NDST enzymes play in HS biosynthesis, only one previous morpholino (MO) knockdown study of an *ndst* homolog in zebrafish has been published. According to Harfouche et al [15], MO knockdown of *ndst1* located on chromosome 21 (named *ndst1a* in the present report), resulted in aberrations in blood flow and vessel formation.

In the present study, we report the occurrence of five highly conserved zebrafish Ndst genes and discuss their evolution. All five genes show distinct temporal and spatial expression pattern during early embryogenesis, suggesting that they are involved in crucial developmental events. We also show that reduced expression of one of the genes, *ndst1b*, results in delayed development, craniofacial cartilage abnormalities as well as shortened body and pectoral fin length.

## Experimental Procedures

### Animals

Zebrafish (*Danio rerio)* AB, WIK, Tg(fli1:GFP) and Tg(1.7col2a1a:mEGFP) strain embryos were obtained by natural spawning and maintained at 28.5°C in E3 medium [16].

### Ethics statement

The protocol was approved by Uppsala Djurförsöksetiska nämnd, Uppsala, Sweden (Permit number C262/11).

### Cloning of zebrafish Ndst cDNA

Mouse NDST protein sequences (Accession id:s NP_032332.2, NP_034941.2, NP_112463.2, NP_072087.1) were used as templates in tblastn searches of the Ensembl *Danio rerio* database (http://www.ensembl.org). Hits with significantly lower E-values and higher sequence similarity to 3-*O*-sulfotransferase-2 than to any mouse NDST were regarded as non-relevant. Using this method, five putative *ndst* zebrafish transcripts were found; *ndst1a*; ENSDART00000085748, *ndst1b*; ENSDART00000090213, *ndst2a*; ENSDART00000125149, *ndst2b*; ENSDART00000085743, *ndst3;* ENSDART00000146084.

Primers were designed to amplify the sequences by PCR using Advantage HD polymerase (Clontech; Primer sequences are available upon request). PCR products were cloned into either pENTR/D-TOPO (Invitrogen) vector or pCRII-TOPO vector (Invitrogen). Plasmid DNA was purified with GeneElute plasmid miniprep kit (Sigma) and sequenced using Big Dye v1.1 (Applied Biosystems). Sequences from at least three separate PCR products of each gene were aligned and analyzed using VectorNTI (Invitrogen). Wherever there was a contradiction between the sequences, the “best of three-principle” was applied.

### Phylogenetic analysis

NDST gene and protein sequences from the following species were collected from the Ensembl database [17] for human (ENSG00000070614, ENSG00000166507, ENSG00000164100, ENSG00000138653); mouse (ENSMUSG00000054008, ENSMUSG00000039308, ENSMUSG00000027977, ENSMUSG00000027971); chicken (ENSGALG00000004581, ENSGALG00000005107, ENSGALG00000012010, ENSGALG00000012015); coelacanth (ENSLACG00000016258, ENSLACG00000006124, ENSLACG00000004348, ENSLACG00000011266); zebrafish (ENSDARG00000074936, ENSDARG00000062397, ENSDARG00000086269, ENSDARG00000060678, ENSDARG00000041776, ENSDARG00000088391); stickleback (ENSGACG00000018023, ENSGACG00000002458, ENSGACG00000018022); lamprey (ENSPMAG00000001193, ENSPMAG00000008519, ENSPMAG00000002673); seq squirt (ENSCING00000005957), drosophila (FBgn0020251) and *C*. *elegans* (F08B4.6).

BLAST searches were performed against the Uniprot database [18] using human NDST sequences as queries. NDST-like sequences were retrieved from lancelet (C3YFU1), purple sea urchin (H3HTR5), starlet sea anemone (A7STS7), *Trichoplax* (B3S367) and sponge, *Amphimedon queenslandica* (I1FI13).

We also performed BLAST searches against the elephant shark, *Callorhinchus milii* genome database [19] and retrieved three NDST-like sequences (AAVX01193879.1; AAVX01040746.1; AAVX01090192.1).

Amino acid sequences were aligned with ClustalX2 [20]. The default alignment parameters were applied. Then the sequences were realigned and bootstrapped 1000 times using SEQBOOT from Phylip 3.695 [21]. Protein distances were calculated using PROTDIST. The Jones–Taylor–Thornton matrix was used for the calculation. The neighbor-joining trees were calculated from the 1000 different distance matrixes, previously generated with PROTDIST, using NEIGHBOR from Phylip 3.695. Majority rule consensus tree was constructed with CONSENSE from the same program package. The trees were plotted using TreeView [22].

### Chromosomal synteny analysis

The chromosomal positions and orientation of NDST3 and NDST4 genes together with the two neighboring genes UGT8 (ceramide UDP-galactosyltransferase) and PRSS12 (neurotrypsin) were retrieved from the Ensembl database. Data were collected for human, mouse, dog, chicken, lizard, frog, coelacanth, zebrafish and stickleback.

### Whole-mount *in situ* hybridization

*In situ* probes corresponding to ~1000 bp of the 3′-terminus of the linearized vector containing either one of the zebrafish *ndst* inserts or *ndst2b* PCR product were labeled using DIG RNA labeling kit (Roche). After DIG labeling, unincorporated nucleotides were removed using RNeasy kit (Qiagen).

*In situ* hybridization was performed as previously described [23], with minor changes to the protocol. Briefly, zebrafish embryos from different developmental stages were dechorionated by pronase treatment, fixed in 4% paraformaldehyde, dehydrated in methanol and stored at −20°C. After thawing, the embryos were washed with phosphate-buffered saline (PBS: 140mM NaCl, 2.7mM KCl, 1.5mM KH_2_PO_4_, 8mM Na_2_HPO_4_^.^2H_2_O) and permeabilized with proteinase K. Hybridization was performed at 67°C with addition of the respective RNA probes in hybridization buffer, after which the embryos were washed once with 2xSSCT buffer and twice with 0.2xSSCT buffer (SSCT is 150mM NaCl, 15mM sodium citrate pH7.0, 0.05%Tween-20). Embryos were then washed with malate buffer (100mM malate pH7.5, 150mM NaCl, 0.05%Tween-20) and incubated in blocking buffer (2% blocking reagent (Roche) in malate buffer). For staining, BM Purple AB Substrate (Roche) was used. After staining, the embryos were washed in PBST (PBS with 0.1% Tween-20), fixed in 4% paraformaldehyde and transferred into 70% glycerol. Photographs were taken using a Leica MZFLIII stereo microscope with a Leica DFC490 digital camera.

Cryosections of the zebrafish embryos were carried as follows; embryos were transferred from a solution of 30% sucrose in PBS to disposable molds (7X7 mm) filled with OCT Cryomount which were frozen in an isopentane bath placed on dry ice. Sectioning was performed at -15°C for the specimens and at -20°C for the blade. The 20 nm sections were collected on glass slides (Superfrost plus) and cleared with Southern Biotech Flourmount-G.

### RT-PCR

Primers designed to amplify zebrafish *ndst1a*, *ndst1b*, *ndst2a*, *ndst2b* and *ndst3* genes were used to perform RT-PCR on cDNA prepared from different zebrafish developmental stages ranging from 2-cell stage up till 50 hpf (primer sequences are available upon request). The PCR reaction (35 cycles) was carried out using AmpliTaq Gold (Roche).

### Morpholino design and injection

Morpholino antisense oligonucleotides (Gene Tools) were designed to target either the *ndst1b* 5’ UTR/translation start (MO1:GCAGGCGGAAAACGCAGAGCATCAC) or intron 5–6/exon 6 junction (MO2: TGATCCTACAGGACAAAACACAACA). Mixture of the standard control morpholino and p53 morpholino oligonucleotides (Gene Tools) were used to exclude off-target effects of the MO injection (referred to as control MO) [24]. Each MO mixture contained 0.2 pmol of p53 MO. The amounts of the MOs used to target *ndst1b* were as follows; 0.1 pmol for MO1 and 0.2 pmol for MO2. Standard control MOs were used at the same concentrations as MO1 and MO2, respectively. As an additional control MO1 and MO2 were used at half of their respective working concentrations.

### Rescue experiments with mouse *Ndst1* mRNA

Full-length *Ndst1* was cloned into the pcDNA3 vector and 5’ capped *Ndst1* was generated by *in vitro* transcription of the pcDNA3 plasmid linearized with SmaI (mMESSAGE mMACHINE T7 kit, Ambion/Invitrogen). Embryos injected with MO1 mixture of morpholinos were divided into two groups. Embryos in the “rescue” group were additionally injected with the 5’ capped *Ndst1*mRNA (22.5 pg) into the yolk at 2–4 cell stage. At 6 dpf, larvae belonging to both morpholino and rescue groups were collected and stained with alcian blue. Severity of the phenotype was quantified. Higher doses of murine *Ndst1*mRNA alone gave unspecific, toxic effect preventing us from using more mRNA to achieve a more efficient “rescue”.

### RT-PCR

To confirm the effect on splicing induced by MO2, 50 zebrafish embryos injected with control MO or MO2 mixtures were collected at either 5 somite stage or 24hpf. Total RNA I kit (E.Z.N.A) was used to purify RNA and the superscript reaction was carried out. Primers specific for *ndst1b* were designed (forward: GCAGGTCAGTGGTCCTCTTC, reverse: AGTCGATGCCACGCTGATAG) and PCR using AmpliTag Gold (Roche) was carried as follows: 95°C-5 min, (95°C-1min, 60°C-20 sec, 72°C-1 min 30 sec) X 35, 72°C-5 min). The control PCR product consisted of 1292 bp, whereas MO injection resulted in an incorrectly spliced product of 1106 bp, confirmed also by sequencing.

### Confocal Imaging of Zebrafish Larvae

Pharyngeal cartilage structures of Tg(fli1:EGFP) and Tg(1.7col2a1a:mEGFP) zebrafish larvae at 6dpf were imaged at 28°C with an inverted Leica SP5 confocal microscope equipped with a heating chamber. Control and morphant larvae were anesthetized and embedded in 1% low melting agarose with 1% Tricaine solution in 35 mm Petri-dishes with a glass cover bottom. Maximum 3D-projections of z-stacks of 25 images were created.

### Alcian blue staining

Alcian blue staining was performed as described previously [25].

## Results

### Identification and phylogenetic analysis of the zebrafish Ndst gene family

**Fig 1:**
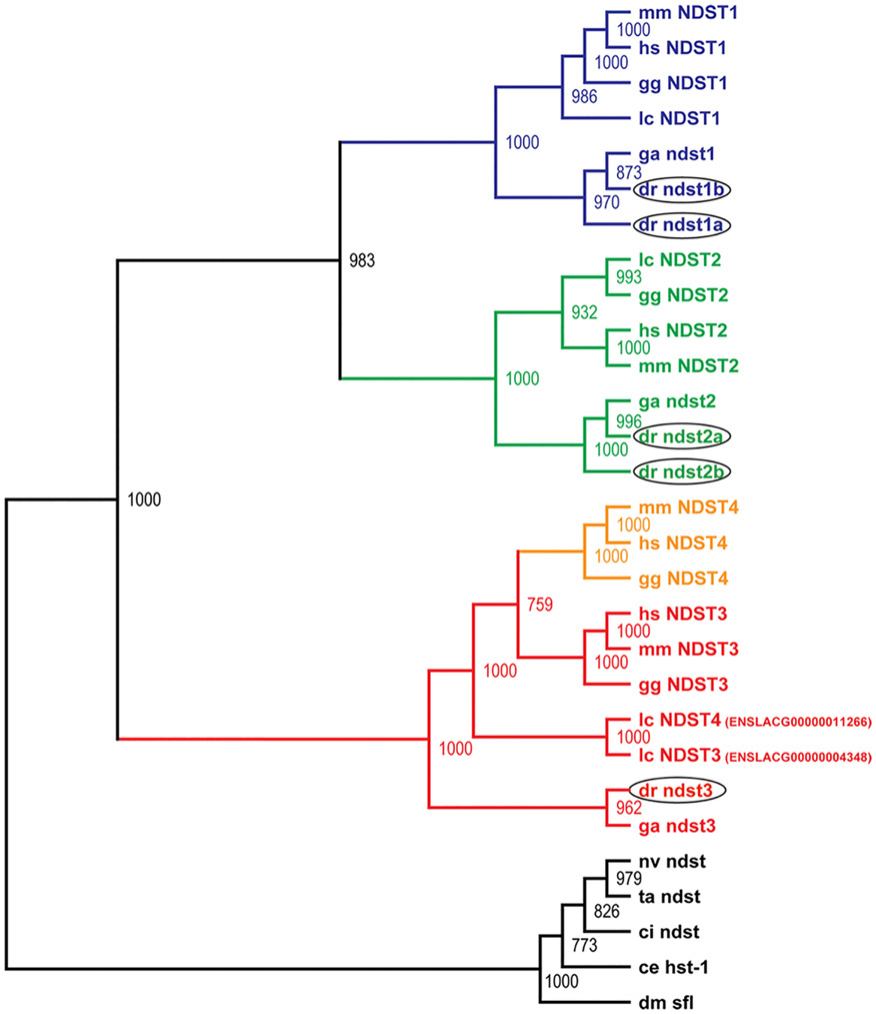
Phylogenetic analysis of the NDST protein family. Based on sequence similarity and phylogenetic analysis, the NDST enzymes can be divided into three main branches: NDST1, NDST2 and a common branch for NDST3 and NDST4. Invertebrate genomes contain a single *ndst*. The consensus neighbor-joining tree was calculated with 1000 bootstrap replicates. Zebrafish Ndst enzymes are encircled. Species names are abbreviated as follows: hs, human; mm, mouse; gg, chicken; ga, stickleback; lc, coelacanth; dr, zebrafish; ci, sea squirt; dm, fruit fly; ce, *C*. *elegans*; nv, starlet sea anemone; ta, *Trichoplax*. The accession numbers are listed under Experimental procedures.

By *in silico* search of the Ensembl database using mouse NDST sequences as templates, five putative zebrafish *ndst* orthologues were identified, cloned and sequenced. Based on amino acid sequence similarity and phylogenetic analysis, the zebrafish Ndst enzymes along with other known vertebrate *NDST* orthologues, form three main subgroups (Fig. 1), including the NDST1, the NDST2 and a common subtype for NDST3 and NDST4. In invertebrates, Ndsts are represented by a single member, sharing 35–56% amino acid identity with vertebrate NDST proteins (Fig. 1; S1 Table). Among the five zebrafish Ndsts, two of the proteins were found to be most similar to the NDST1s and were named Ndst1a and Ndst1b, while two of the others showed most similarity to the NDST2s and were named Ndst2a and Ndst2b. Comparing the amino acid sequences, zebrafish Ndst1b has slightly lower identity to other vertebrate NDST1 enzymes than Ndst1a (65–74% amino acid sequence identity compared to 73–81%). Similarly, zebrafish Ndst2b shares 73–79% identity to other NDST2 protein sequences compared to 76–86% for Ndst2a (S1 Table). The fifth zebrafish Ndst is found in the common NDST3/NDST4 subgroup (Fig. 1; S1 Table) and it shares 74–77% identity with tetrapod Ndst3 and Ndst4 amino acid sequences.

### *ndst3* or *ndst4*?

**Fig 2:**
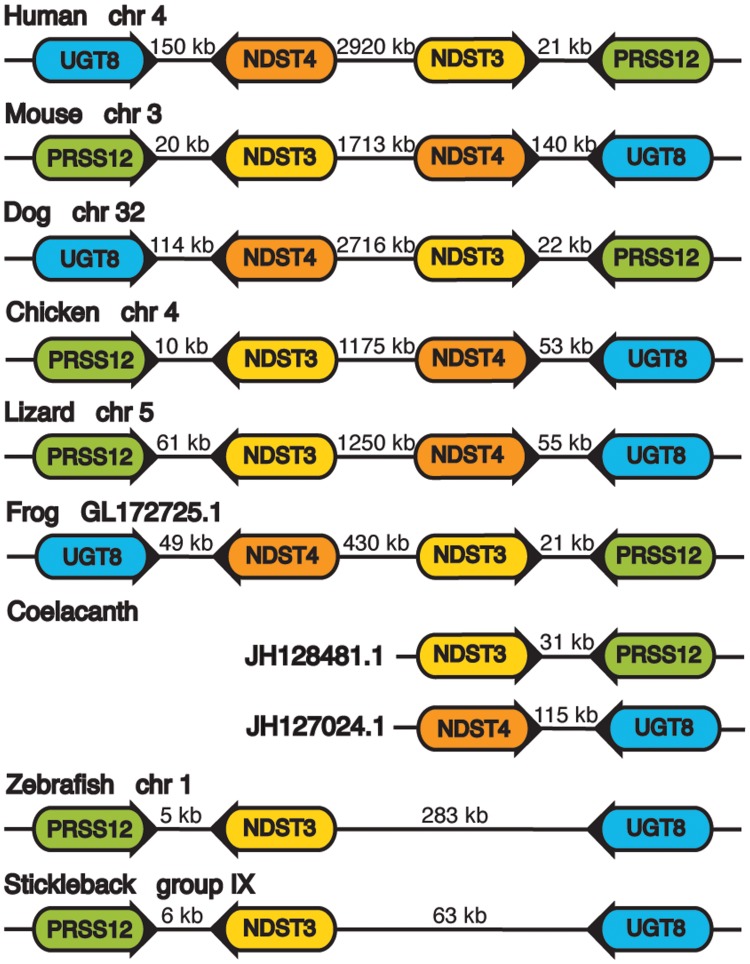
Schematic presentation of chromosomal or scaffold position of *NDST3*, *NDST4*, *UGT8* and *PRSS12* in different vertebrate species. Arrowed rounded rectangles display the orientation of the genes. Numbers between the genes indicate the distances in kilo base pairs. Gene name abbreviations: *UGT8* (ceramide UDP-galactosyltransferase), *PRSS12* (neurotrypsin).

To elucidate the relation of the single zebrafish orthologue to *NDST3* and *NDST4*, we analyzed *NDST3* and *NDST4* localization and orientation together with the neighboring genes encoding ceramide UDP-galactosyltransferase (*UGT8*) and neurotrypsin (*PRSS12*) in different genomes (Fig. 2). Notably, in vertebrates *NDST3* and *NDST4* always have opposite orientation, where *NDST3* is facing *PRSS12* and *NDST4* is facing *UGT8* with 20–150kb between their 3'-ends (Fig. 2). In tetrapods, NDST3 and NDST4 subtypes share around 80% identity in amino acid sequence and are more similar to each other than to other NDST subtypes.

Also coelacanths contain two genes orthologous to the *NDST3* and *NDST4* types, while cartilaginous and bony fishes only have one (Fig. 1). Furthermore, location and orientation of the coelacanth *ndst3* and *ndst4* genes are similar to those of the tetrapods. Thus, it is likely that a local duplication in the common ancestor of lobe-finned fishes and tetrapods gave rise to the two genes. In zebrafish and stickleback, the single *ndst* orthologue is facing *prss12* with only 5–6 kb between their 3'-ends (S1 Fig.). The orientation and position of this gene in zebrafish and stickleback is the same as the *NDST3* gene in other vertebrates (Fig. 2). Therefore, we named this gene *ndst3*.

### Zebrafish Ndsts resemble their vertebrate orthologues

Similar to all NDST isoforms found in other vertebrates, the amino acid sequence of zebrafish Ndst proteins differ mostly in the N-terminal part of the proteins (S1 Fig.). The N-terminal end consists of a short cytoplasmic domain, around 20 amino acids long, followed by a stretch of highly hydrophobic residues, representing a transmembrane spanning domain [28].

The NDST domain responsible for the *N*-sulfotransferase activity is located in the carboxyl half of the protein, previously demonstrated by the expression of this part of the mouse NDST2 as an active *N*-sulfotransferase [29]. The corresponding domain from human NDST1 has been crystallized [28] and used for modeling of mouse and human NDST structure [30], revealing differences between the isoforms in the putative HS substrate binding cleft. However, the motifs for the 5′- and 3′-PAPS binding sites [27] are well conserved among the isoforms. This applied also to the five Ndsts found in zebrafish (S1 Fig.). The *N*-deacetylase domain has so far not been crystallized, but is present in a A66-P604 fragment of human NDST2, expressed with retained *N*-deacetylase activity in *E*. *coli* [26]. As first demonstrated for rat NDST1 [31] and later for murine NDST1 [32], the cysteine at position 486 (corresponding amino acids marked with an asterisk in S1 Fig.) is close to the active site, as mutation of this residue to an amino acid with a large side chain abolishes *N*-deacetylase activity, while replacing it with alanine or valine results in enhanced enzyme activity. In all human, mouse and zebrafish NDST proteins this cysteine residue is conserved.

### Expression of five different zebrafish *ndst* mRNA

**Fig 3:**
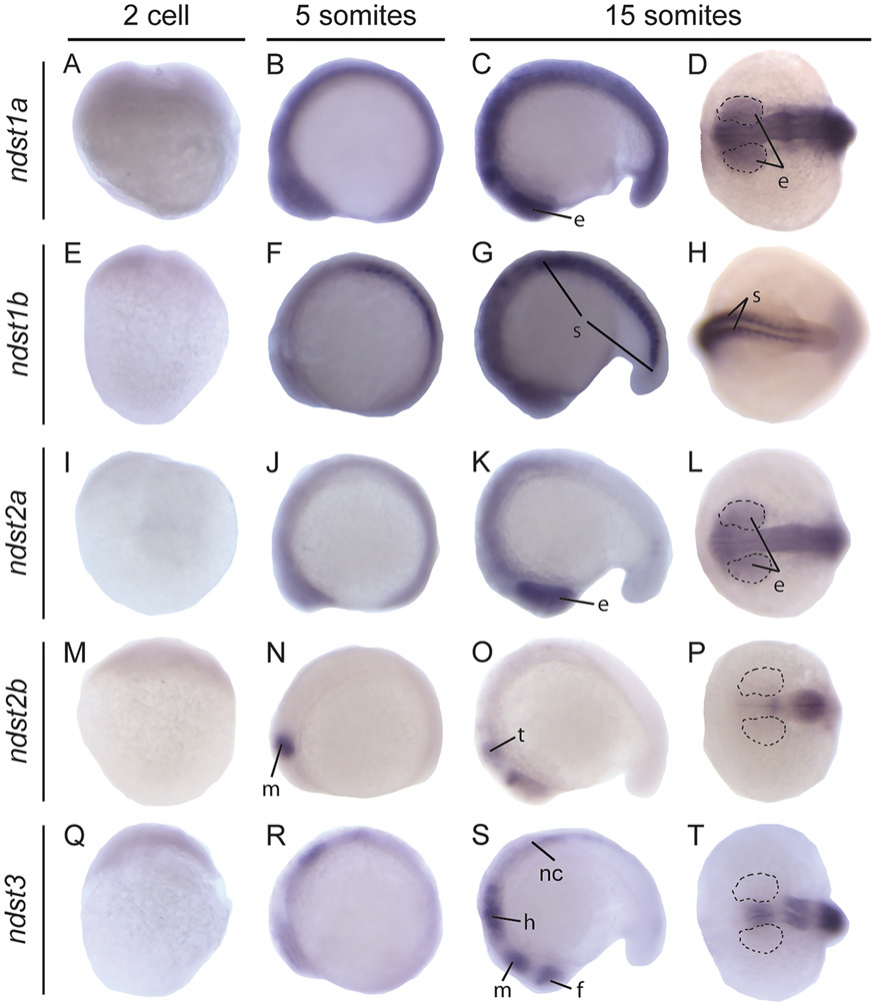
Expression of zebrafish *ndst* genes at early developmental stages. Lateral view of embryos at 2 cell, 5 somite and lateral and dorsal view of embryo head (D, L, P, T) or tail at 15 somite stage, respectively. Expression of *ndst1a*: A-D; *ndst1b*: E-H; *ndst2a*: I-L; *ndst2b*: M-P; *ndst3*: Q-T. Areas of the developing eye are marked with dotted lines. Abbreviations used: e, eye; f, forebrain; h, hindbrain; m, midbrain; nc, neural crest; s, somites; t, tectum.

To study the distribution of the *ndst*s, *in situ* hybridization was performed at various embryonic stages, from the 2 cell stage until 3 days post fertilization (dpf), with probes specific for each of the five zebrafish *ndst* mRNAs (Table 1). All isoforms were maternally contributed as shown by staining and RT-PCR at the 2 cell stage (Fig. 3A, E, I, M, Q; S2 Fig.), a time point at which zygotic transcription has not yet commenced. All *ndst* genes showed distinct expression patterns, indicating that the *in situ* hybridization probes did not cross-hybridize with other *ndst* family members.

**Table 1:** Expression of *ndst* genes in zebrafish embryos during early development.

	*ndst1a*	*ndst1b*	*ndst2a*	*ndst2b*	*ndst3*
Maternal	+	+	+	+	+
Notochord	-	-	-	-	-
Floor plate	-	-	-	-	-
Hypochord	-	-	-	-	-
Somites	+	++	+	-	-
Eye	+	+	+	+	-
Olfactory system	+	-	+	+	++
Otic capsule	+	+	+	+	+
Posterior ICM	-	+	-	-	-
Branchial arches	+	+	+	+	-
Pectoral fin	++	+	+	+	++
CNS					
Telencephalon	+	-	+	+	++
Diencephalon	+	-	+	+	+
Midbrain-Hindbrain boundary	++	+	+	+	++
Midbrain	+	+	++	+	++
Hindbrain	+	++	+	+	++
Spinal cord	-	-	-	-	++

**Fig 4:**
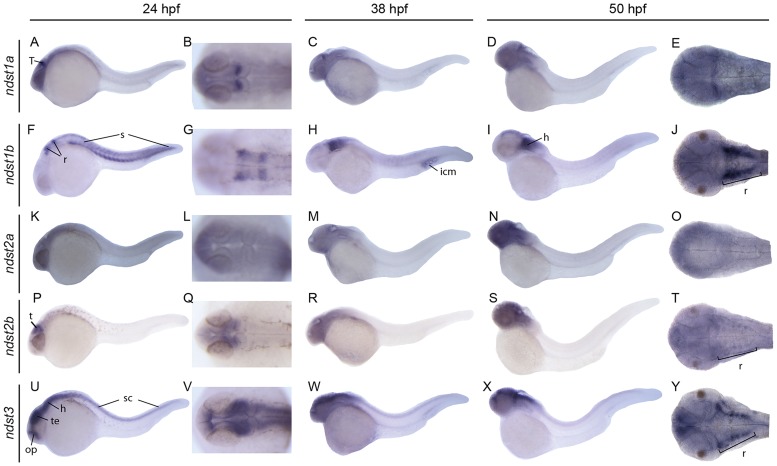
Expression of zebrafish *ndst* genes at 24, 38 and 50 hpf. Lateral and dorsal view of the embryos, or head region of the embryo. Embryonic expression patterns are shown for *ndst1a*: A-E; *ndst1b*: F-J; *ndst2a*: K-O; *ndst2b*: P-T; *ndst3*: U-Y. Abbreviations used: h, hindbrain; icm, intermediate cell mass (blood islands); op, olfactory placode and olfactory bulb; r, rhombomeres; s:, somites; sc, spinal cord; t, tectum; te, telencephalon.

Each of the probes showed unique staining in the head region throughout investigated developmental stages. However, only three of the genes were transcribed in the trunk at 24 hours post fertilization (hpf), more specifically in the spinal cord (*ndst3*) and in the somites (*ndst1a* and *ndst1b*) (Fig. 4). *ndst3* expression in the spinal cord was persisting also at 50 hpf whereas myotomal staining of *ndst1b* was very weak at this time point (Fig. 4).

**Fig 5:**
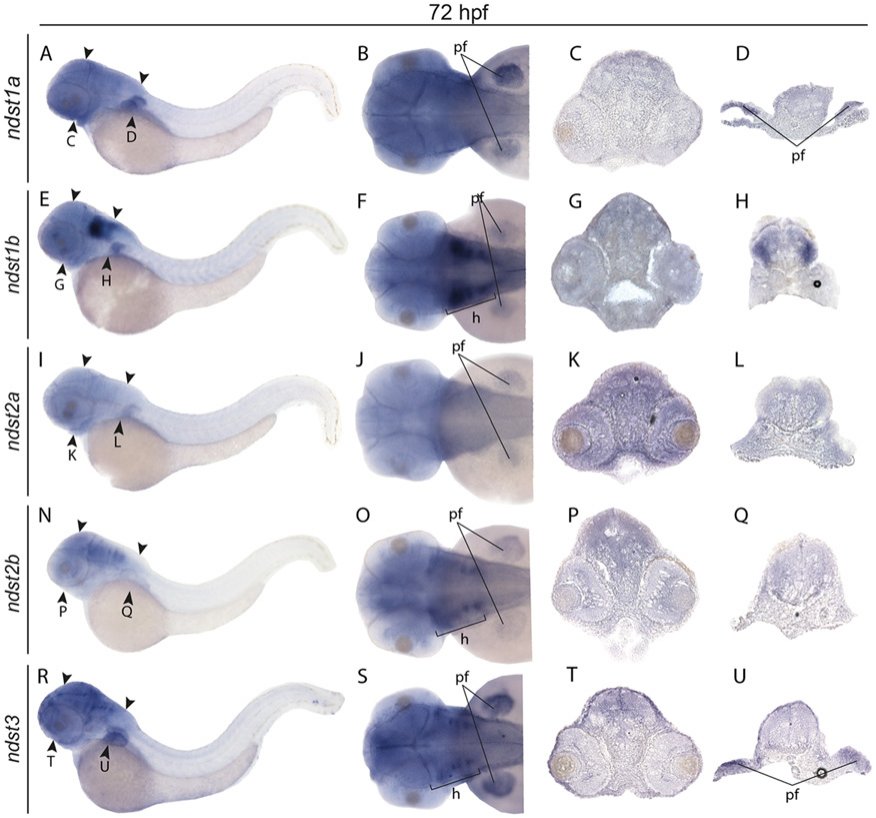
Expression of zebrafish *ndst* genes at 72 hpf. Lateral views of larvae (first column to the left), dorsal views of larval heads (second column to the left) and transversal sections showing the expression patterns of *ndst1a*: A-D; *ndst1b*: E-H; *ndst2a*: I-L; *ndst2b*: N-Q; and *ndst3*: R-U. Positions of the sections are indicated with arrow heads. Abbreviations used: h, hindbrain; pf, pectoral fins.

*ndst1a* showed an ubiquitous expression pattern at early stages (Fig. 3A-D), whereas at 24 hpf expression was restricted mostly to the posterior part of optic tectum (Fig. 4A-B), persistent also at 38 and 50 hpf (Fig. 4C-E). At 72 hpf weak staining was observed in the head region as well as in the developing pectoral fins (Fig. 5A-D) *ndst1b* was the main isoform expressed in the somites already at the 5 somite stage (Fig. 3F) and was later peaking in the myotome at 24 hpf (Fig. 4F). At 24 hpf, *ndst1b* expression was observed in rhombomeres and weaker staining was seen in the region of the migratory neural crest (Fig. 4F-G). At 50 hpf expression was observed in brain tissues, showing specific staining in hindbrain and along the borders of the optic tectum (Fig. 4I-J). The same trend was observed at 72 hpf, with *ndst1b* dominant expression in the rhombomeres and weak expression in the pectoral fins (Fig. 5E-H).

During early somitogenesis, *ndst2a* expression was seen in the entire embryo (Fig. 3I-L), with staining prevailing at the 15 somite stage in the anterior part of the embryo and in the developing eye (Fig. 3K-L). Between 24 hpf and 72 hpf, *ndst2a* expression was localized to the head region of the embryo (Fig. 4K-O, Fig. 5I-L). In contrast, *ndst2b* showed a very specific expression pattern already at the 5 somite stage when staining was restricted to the developing midbrain (Fig. 3N). At the 15 somite and 24 hpf stage, expression was detected in the tectum (Fig. 3O-P, Fig. 5P-Q), whereas at 50 and 72 hpf most of the brain tissues were stained, although more intensely in the rhombomeres (Fig. 4S-T, Fig. 5 N-Q).

During early somitogenesis, *ndst3* was expressed in all brain parts including forebrain, midbrain and the hindbrain (Fig. 3R-T), with additional staining of spinal cord at 24 hpf (Fig. 4U), persistent through all investigated stages of development, until 72 hpf (Fig. 4U-Y, Fig. 5R-U). Additionally *ndst3* was expressed in the pectoral fins and weakly in the dermis (Fig. 5 R-U).

### Ndst1b is needed for correct cranial cartilage formation

To decipher the functions of the different Ndst enzymes during development, pilot-experiments with morpholino knockdown targeting the five *ndst* isoforms, one at a time, have been performed (results not shown). However, so far only *ndst1b* morpholino injections have resulted in a stable phenotype, most likely due to the overlapping expression of the other *ndst* isoforms.

**Fig 6:**
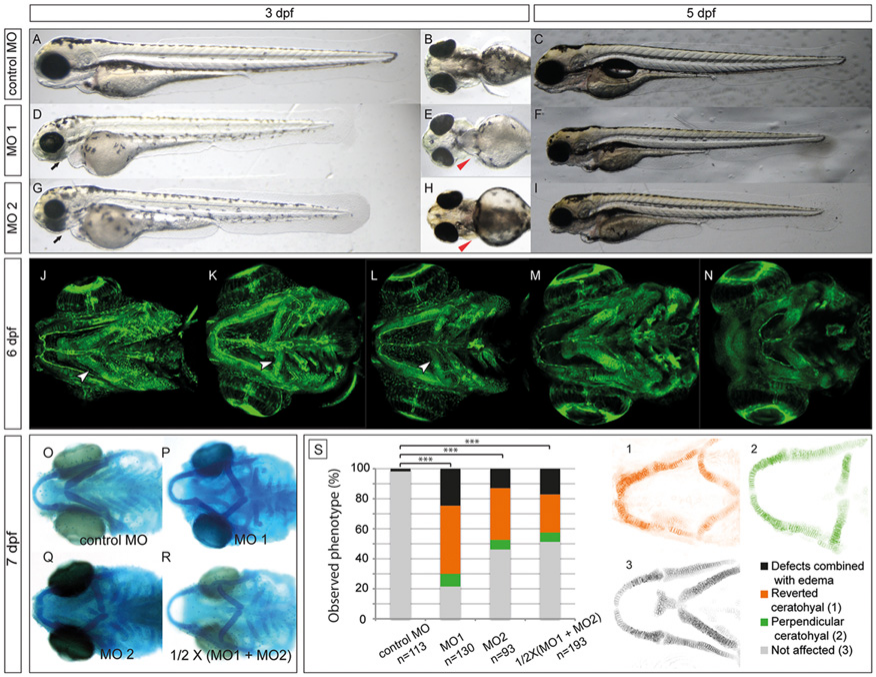
Morpholino knockdown of zebrafish *ndst1b* results in craniofacial cartilage defects at the early stages of development (3–7 dpf). Lateral and ventral view of a zebrafish larvae at 3 dpf injected with either control MO mix (A-B), MO1 (D-E) or MO2 (G-H) in combination with p53 MO (see Experimental Procedures). The black arrows point at the underdeveloped craniofacial cartilage of *ndst1b* morphants, while the red arrow heads point at the pectoral fins of the morphants, shortened in comparison to fins of the control larvae. At 5 dpf, the effects of the *ndst1b* MOs on zebrafish larvae were still visible. *ndst1b* MO treated larvae had shortened pectoral fins and body axis, smaller head and eyes, underdeveloped craniofacial cartilage and no swimming bladder (C,F,I). Confocal microscope images present a ventral view of Tg(fli1:EGFP) zebrafish larvae. GFP is expressed in endothelial and neural crest derived cells. Morphants displayed severe malformations in the pharyngeal cartilage structures (K, L) in comparison to control larvae (J). In contrast, chondrocyte stacking and flattening does not appear to be strongly affected, as compared to *extl3* ^*-/-*^ and *ext2* ^*-/-*^ mutant embryos (M-N) with clear chondrocyte stacking defects. The second pharyngeal arch (the ceratohyal), incorrectly localized in the *ndst1b* morphants, is indicated with a white arrow head (J-L). The incorrect localization is also seen after alcian blue staining of 7 dpf larvae injected with MO1, MO2 and half working dosage of these two combined (1/2 X (MO1 + MO2); O-R). Panel S of the figure represents quantification in percentage of the ceratohyal phenotype at day 6 of the development in phenotypic classes observed (chi-square test: *** p<0.001 comparing affected and nonaffected embryos). Confocal microscope images of the Tg(1.7col2a1a:mEGFP) fish (1–3) show the difference between reverted (1), perpendicular (2) and non-affected ceratohyals.

At 6 dpf, approximately 80% of the *ndst1b* morphant larvae injected with either translational blocking (MO1), or 55% of the ones injected with splice blocking mix (MO2) exhibited a distinct, uniform phenotype (Fig. 6S). Exon 6 was chosen as a splice MO target (MO2) since it contains the cysteine residue shown to be critical for *N*-deacetylase activity (S3 Fig.) [31, 32]. In addition control co-injection of a half working-concentration of MO1 and MO2 resulted in 50% penetrance and a similar phenotype as injection of MO1 or MO2 alone (Fig. 6R-S). For each of the performed morpholino injections, MO targeting *p53* was included to avoid the off-target *p53* up-regulation effect [24]. To confirm the specificity of the splice MO knockdown (MO2), RT-PCR was performed with cDNA synthesized from mRNA isolated from 24 hpf embryos. The obtained products matched the predicted lengths of the PCR products with and without exon 6 in the template mRNA. Their identities were also confirmed by sequencing of the PCR product (S3C Fig.).

At 3 dpf and 5 dpf injected larvae had slightly shortened body length compared to controls, smaller eyes and disorganized pigment-cell patterning (Fig. 6A-I). At 6 dpf injected larvae were 4% shorter (p<0.0001 using t-Test on body lengths of 27 control and morphant larvae). Most of the affected *ndst1b* morphants also had shortened pectoral fins, a characteristic feature of previously described glycosaminoglycan mutants (Fig. 6B, E, H) [33, 34]. Confocal images of 6 dpf zebrafish Tg(fli1:GFP) transgenic larvae injected with either MO1 or MO2 (in combination with *p53* MO) revealed incorrect localization of the ceratohyal (Fig. 6J-L) as well as severely shortened/missing basihyal and branchial arches with a penetrance reaching at least 50% (Fig. 6K-L), in contrast to larvae injected with control morpholino mixture (Fig. 6J).

The specificity of the morphant phenotype was challenged in rescue experiments performed on MO1 injected embryos with mouse *Ndst1* mRNA resulting in a reduction of the proportion and severity of the morpholino phenotypes (S4 Fig.). Unfortunately, we could not inject more of rescuing mRNA to achieve a better penetrance, since increasing concentrations of mNDST1 mRNA alone resulted in aberrant phenotypes (most likely due to heparan sulfate oversulfation). Applying statistics to the data, one of the experiments shows significant difference between affected and unaffected embryos (p = 0.04), while the p-value for the second experiment is 0.14. However, also in this experiment the number of affected embryos is larger than that of unaffected. Together with the fact that the morpholino phenotype is rare and that the two different morpholinos and a combination of half-working concentration of both morpholinos give the same phenotype, we feel convinced that the morpholino phenotype is not caused by off-target effects.

Interestingly, cartilage structures forming the first pharyngeal arch, (Meckel’s cartilage, palatoquadrate and hyosymplectic) appear to be morphologically normal. In contrast, *extl3* ^*-/-*^ and *ext2* ^*-/-*^ larvae display a clear chondrocyte stacking defect in the same structures (Fig. 6J-N). The lack of the chondrocyte stacking phenotype was also confirmed in 7 dpf larvae by alcian blue staining (Fig. 6O-R), which at low pH depends on the sulfate substitution of glycans [35]. In the zebrafish larvae, alcian blue staining overlaps with sites of chondroitin sulfate accumulation, such as cartilage elements [25]. In *ndst1b* morphants no difference in the intensity of cartilage staining was seen in comparison to controls (Fig. 6O-R), indicating that chondroitin sulfate biosynthesis and therefore Golgi apparatus function was not strongly affected in the morphants.

Higher doses of the MO targeting *ndst1b* resulted in strong developmental malformations, including extensive edema, body axis malformations, as well as nearly complete blockage of craniofacial cartilage formation (data not shown). These results may point to a general need of *ndst1b* expression in the zebrafish development, but could also be explained by toxic effects of high doses of injected morpholino.

## Discussion

All modification enzymes involved in HS biosynthesis in zebrafish, except for the Ndst protein family, have previously been described [9–14]. In this study, five zebrafish Ndst family members with differential expression and highly conserved structural domains important for catalytic activities are reported (S1 Fig.).

The protein sequence identity of mammalian NDST orthologues is more than 90%, and that of zebrafish and mammalian NDSTs is higher than 70% (S1 Table). The genome of all investigated invertebrates contain a single Ndst gene, where the resulting protein shares up to 56% sequence identity with vertebrate homologues (Fig. 1). The invertebrate Ndst proteins all contain conserved residues within the *N*-deacetylase domain as well as within the 5'-phosphosulfate (5'PSB) and 3'-phosphate binding (3'PB) motif of the *N*-sulfotransferase domain (S1 Fig.). The previously reported residue Lys 614, critical for *N*-sulfotransferase activity of human NDST1 [36], is part of a GPQK motif conserved in all species. Also the cysteine residue, important for *N*-deacetylase activity in mammalian enzymes [31] is part of a motif, LPRQTC, conserved in all vertebrate and invertebrate species.

The presence of the Ndst protein in Trichoplax, the structurally simplest multicellular animal (Fig. 1, S1 Table), indicates that the NDST family appeared in the common ancestor of all metazoans and that only a single NDST enzyme was needed in these organisms until the divergence of vertebrates. According to the 2R hypothesis [37], the genomes of the early vertebrate lineage underwent two rounds of genome duplication [38]. The presence of duplicated orthologues of *NDST1* and *NDST2* in zebrafish is an indicator of the previously proposed, additional third round of teleost specific genome duplication [39]. The orientation of tetrapod genes *NDST3* and *NDST4* in relation to each other and the neighboring genes *PRSS12* and *UGT8* is conserved in all species (Fig. 2). The close proximity of *NDST3* and *NDST4* supports the notion that *NDST4* most likely arose through a local duplication of *NDST3* before the divergence of coelacanth from the lineage leading to tetrapods. The outward orientation of the *NDST3* and *NDST4* genes may suggest that the region between the two genes has an important regulatory function. Intriguingly, this region in the human, mouse, lizard and also in the zebrafish genome contains long intergenic non-coding RNA and small nuclear RNA. Tentatively, the region contains regulatory elements for both *NDST3* and *NDST4* and possibly also for UGT8 and *PRSS12* transcription.

Both during embryonic development and in adult mice, *NDST1* and *NDST2* transcripts are present in most tissues, whereas *NDST3* and *NDST4* are mostly expressed during embryonic development, *NDST3* also found in adult brain [30, 40]. NDST1 *in situ* hybridization and immunostaining of mouse embryo sections show expression primarily in brain and in distal limb structures [41]. In zebrafish, all *ndst* enzyme isoforms were expressed in varying brain regions throughout the development, with the *ndst3* isoform expressed predominantly in the central nervous system. Previous studies of the other HS modifying enzymes also revealed highly specific spatial and temporal expression patterns of enzyme paralogs during *Danio rerio* development [12, 42–46] indicating a need of strict regulation of HS structure in developmental processes. Differences in enzyme kinetics and substrate preferences of the different isoforms could possibly result in variable HS structures. Along this line, the four mouse NDSTs were shown to exhibit differing kinetics [30]. Further, *in vivo* studies of NDST transgenic mice have contributed to the understanding of the roles of the different isoforms. Mice deficient in NDST2 produce HS with the same structure as control mice, are viable and fertile, but their mast cells are devoid of heparin, suggesting that the main function of NDST2 is in the biosynthesis of this highly sulfated molecule [47, 48]. However, NDST2 also appears to be important for HS biosynthesis in other cells than mast cells since embryos deficient in both NDST1 and NDST2 die during early embryogenesis [49]. A study on NDST3 knockout mice reported no major defects, but lack of both NDST1 and NDST3 results in a somewhat more severe phenotype than that of NDST1 alone [50]. NDST4 mouse mutants are so far not available.

Mice deficient in NDST1 die shortly after birth, due to respiratory failure [51, 52]. Mutant embryos have severe facial defects in the regions where *Ndst1* is normally expressed, including forebrain, eyes and neural crest derived facial structures [41, 53, 54]. HS isolated from organs of NDST1^-^/^-^ mice embryos at embryonic day 18.5 has reduced *N*-sulfation levels and a 50% decrease in overall sulfation compared to wild type embryos [2, 51]. In the recent study by Reuter et al., four families with missense mutations in the NDST1 *N*-sulfotransferase domain were identified. Patients affected by these mutations exhibit autosomal recessive intellectual disability, epilepsy, deficiency in postnatal growth and muscle hypotonia [55, 56].

In zebrafish, *ndst1b* and to a somewhat lower extent *ndst1a* were expressed in the somites, which give rise to muscle segments and axial skeleton. However, *ndst1b* morpholino (MO) knockdown did not result in any pronounced muscle phenotype, indicating that Ndst1a expression in zebrafish is sufficient for muscle development. Zebrafish *ndst1b* morphants instead displayed an altered chromatophore (pigmented cells) distribution. In addition, cartilaginous elements in the second pharyngeal arch and in the branchial arches were affected, indicating a role for *ndst1b* in neural crest subpopulation cell fate [57].

Zebrafish mutants for *papst1*, *ext2*, *uxs1* and *b3gat3*, all related to GAG biosynthesis [33, 34], display a strong cartilage phenotype characterized by defective chondrocyte flattening and intercalation resulting in thick and short pharyngeal cartilage elements. All arches in these mutants are affected in a similar manner leading to a anterior-posterior axis compression. The chondrocytes in the skeletal elements of the *ndst1b* morphants did not exhibit such severe defects. Instead, the orientation of the elements is altered. The ceratohyal and the branchial arches are located perpendicular to the basihyal as opposed to their normal oblique orientation seen in the wild-type larvae. Interestingly, the first pharyngeal arch is not affected and was formed correctly. Similar craniofacial phenotypes as those reported in our study have been reported for the *moz* and *mob* mutants as well as for the *esco2* morphant [58–60], but are otherwise rare. These observations may suggest that intercalation of chondrocytes within the cartilage elements and orientation of the arches are dependent on separate mechanisms. A possible explanation of the perpendicular orientation of the ceratohyal is that the second pharyngeal arch and the branchial arches follow the extension of the basihyal, and since the basihyal is extremely shorter or missing, the arches 2–7 cannot grow properly. Although *ndst1b* in situ hybridization did not reveal any obvious expression of the gene in the craniofacial cartilage, *ndst1b* transcript was detected at 24, 38, 50 hpf and 72 hpf in the rhombomers of the hindbrain, important for correct formation of the pharyngeal arches. Tentatively, this process is less sensitive to decreased NDST activity than migration of the neural crest cells. A role for NDST1 in neural crest cell fate was previously suggested based on the severe skull malformations observed in mouse NDST1^-/-^ embryos [41]. In a recent report from the Grobe lab, it was demonstrated that expression of *Ndst1* in the neural crest also is important for mouse cardiogenesis [61]. Additionally, the expression pattern of *ndst1b* at 24 hpf strongly resembles *hoxa2b in situ* staining, with additional similarities between *ndst1b* and *hox2* morphant fish. This result strengthens our hypothesis that *ndst1b* plays an important role in craniofacial primodia, similar to *hox2b*, a marker of pharyngeal arch development [60].

A few functional studies on HS biosynthetic enzymes in zebrafish using the MO knockdown technique have been published. These studies suggest that 6-OST-2 is involved in muscle and vascular development [12, 44] and that the C5-epimerases are important for dorso-ventral axis formation [43]. Morpholino knock down of the 3-OST-5 and 3-OST-6 reveled their importance in cilia length and motility, respectively, and therefore in left-right patterning of the embryo, whereas 3-OST-7 was reported to be crucial for cardiac ventricle contraction [62, 63]. Studies on zebrafish HS polymerase mutants exostosin 2 (*ext2/dackel*) and exostosin like 3 (*extl3/boxer*) have demonstrated the importance of these genes for axon guidance, pectoral fin development as well as craniofacial cartilage development [33, 64, 65]. In this report we have shown that lowered *ndst1b* gene expression results in a reduced body length, smaller eyes and disorganized pigment-cell patterning, shortened pectoral fins and disturbed craniofacial development. In addition, we have demonstrated that the Ndst family in zebrafish has five members, the duplicated *NDST1* orthologues *ndst1a* and *ndst1b*, the duplicated *NDST2* orthologues *ndst2a* and *ndst2b*, and the single orthologue *ndst3*, equally similar to mammalian *NDST3* and *NDST4*. Our future studies will involve functional studies on the different Ndst isoforms. In particular, it will be interesting to investigate the functional consequences of zebrafish Ndst3 deficiency, which would equal an NDST3/NDST4 double knockout in mouse.

## Supporting Information

S1 Fig Multiple alignment of NDST amino acid sequences from human (hs), zebrafish (dr) and fruit fly (dm). The transmembrane region, the *N*-deacetylase domain (corresponding to the truncated protein expressed by Duncan et al. [26] and the PAPS-binding motifs (5’-PSB and 3’-PB as described in Kakuta et al., [27] of the sulfotransferase domain are indicated. The conserved cysteine residue located close to the *N*-deacetylase active site is marked with an asterisk (*).
(TIF)

S2 Fig Temporal mRNA expression of *ndst* genes during early embryonic development. RT-PCR was performed for all *ndst* genes at the indicated stages of development. *β-actin* expression was used as a control.
(TIF)

S3 Fig The *ndst1b* morpholino oligos. (A) The genomic structure of the *ndst1b* gene with the regions targeted by morpholinos MO1 and MO2 in red and primers used for RT-PCR marked by black arrows (forward, F; reverse, R). (B) Structural relationship between the *ndst1b* exons and enzymatic domains. Missing part of the *N*-deacetylase domain as a consequence of MO2 injection is marked with the black arrow head (WT, wild type; MO2, morpholino 2). (C) RT-PCR of 5-somite and 24 hpf embryo RNA confirmed the generation of an *ndst1b* transcript lacking exon 6 in embryos injected with two different doses of the splice morpholino (MO2).
(TIF)

S4 Fig Translational blocking *ndst1b* morpholino phenotype rescue with mouse *Ndst1* mRNA at 6 dpf. Embryos at 1–2 cell stages were injected with MO1 mixture and divided into two groups. Embryos in the “rescue” group were assigned to a second round of injection with *Ndst1b* mRNA. Both the proportion and severity of ceratohyal defects decreased when the larvae were injected with 22.5pg mouse *Ndst1* mRNA. Larvae were classified as not affected (grey), or depending on increasing levels of defects in the second pharyngeal arch as perpendicular ceratohyal (green), reverted ceratohyal (orange) or those with cartilaginous defects combined with edema (black). For classification see Fig. 6. The phenotypic difference beteween embryos injected with morpolino only and those injected with morpholino and mouse *Ndst1 mRNA* is significant in the first experiment (* p<0.05, chi-square test, comparing affected and nonaffected embryos).
(TIF)

S1 Table Amino acid identities (%) between NDST proteins. Species names are abbreviated as follows: hs, human; mm, mouse; gg, chicken; lc, coelacanth; ga, stickleback; dr, zebrafish; ci, sea squirt; dm, fruit fly; ce, *C*. *elegans*; nv, starlet sea anemone; ta, *Trichoplax*. The accession numbers are listed under Experimental procedures.
(TIF)
