# Supplementary figures and images for: The Ndst Gene Family in Zebrafish: Role of Ndst1b in Pharyngeal Arch Formation
Source: PLoS One. 2015 Mar 13;10(3):e0119040. doi: 10.1371/journal.pone.0119040 (PMC4359090; doi:10.1371/journal.pone.0119040)

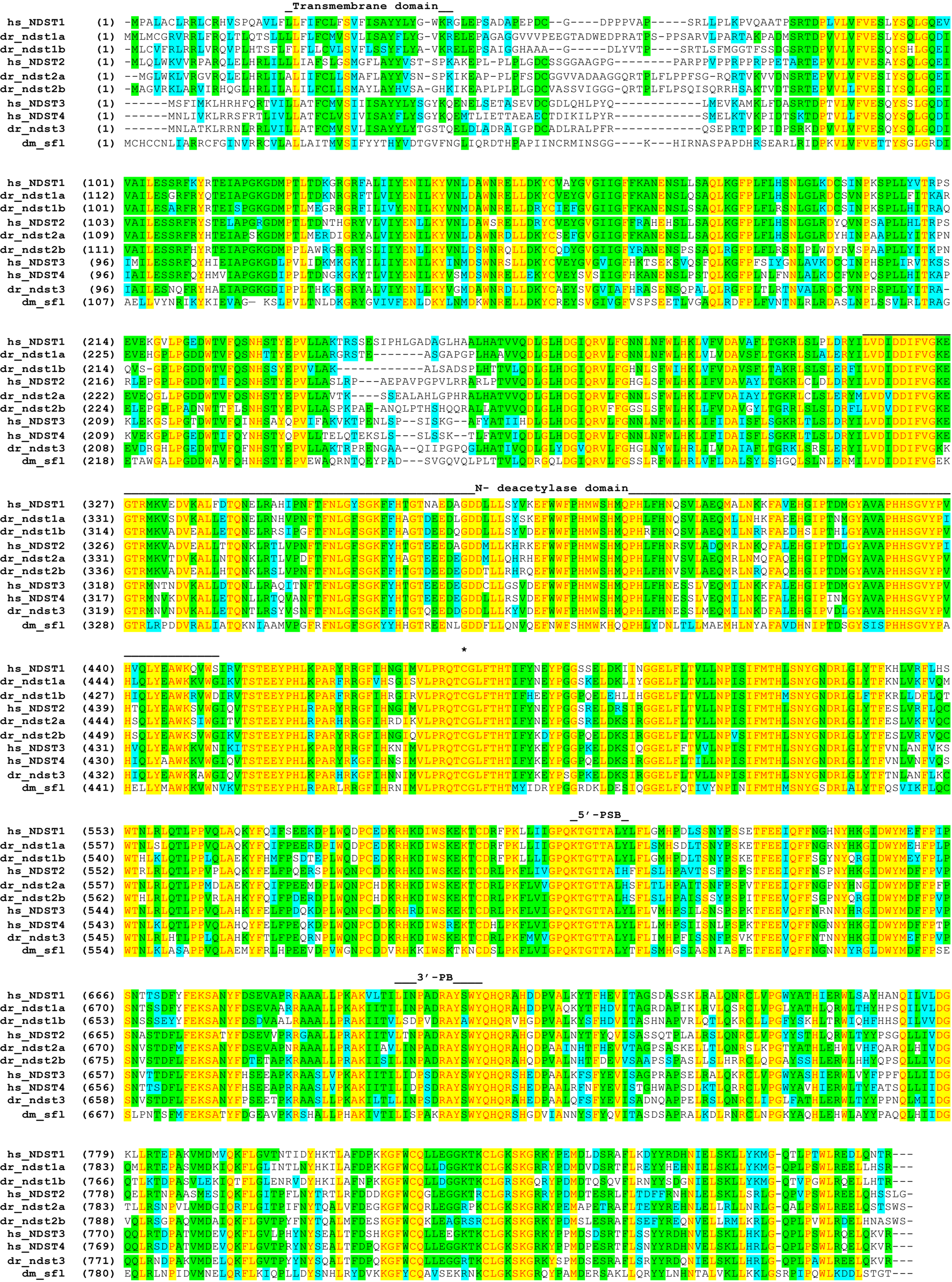

Supplement: S1 Fig — The transmembrane region, the N-deacetylase domain (corresponding to the truncated protein expressed by Duncan et al. [26] and the PAPS-binding motifs (5’-PSB and 3’-PB as described in Kakuta et al., [27] of the sulfotransferase domain are indicated. The conserved cysteine residue located close to the N-deacetylase active site is marked with an asterisk (*). (TIF) [file pone.0119040.s001.tif]

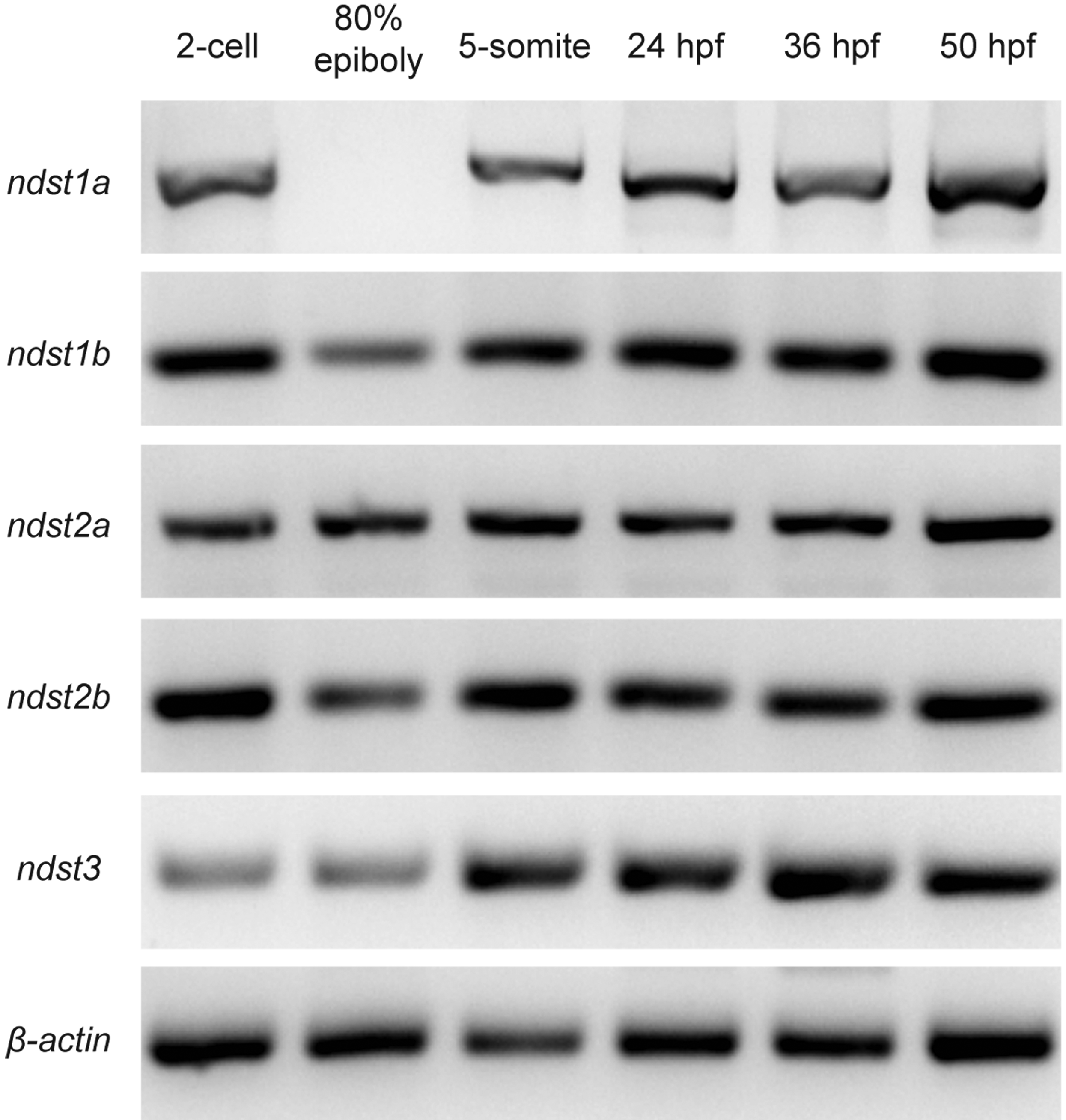

Supplement: S2 Fig — RT-PCR was performed for all ndst genes at the indicated stages of development. β-actin expression was used as a control. (TIF) [file pone.0119040.s002.tif]

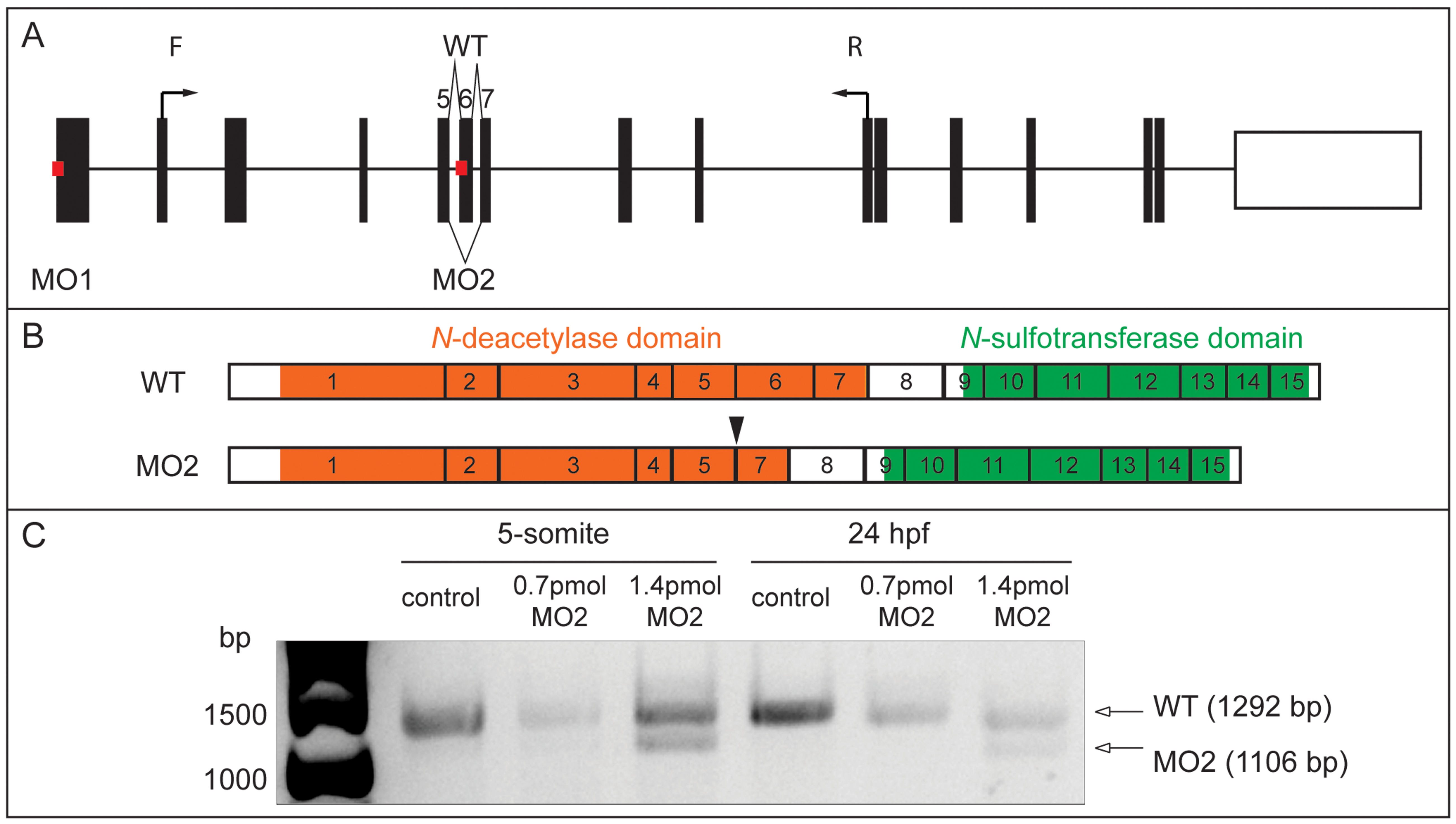

Supplement: S3 Fig — (A) The genomic structure of the ndst1b gene with the regions targeted by morpholinos MO1 and MO2 in red and primers used for RT-PCR marked by black arrows (forward, F; reverse, R). (B) Structural relationship between the ndst1b exons and enzymatic domains. Missing part of the N-deacetylase domain as a consequence of MO2 injection is marked with the black arrow head (WT, wild type; MO2, morpholino 2). (C) RT-PCR of 5-somite and 24 hpf embryo RNA confirmed the generation of an ndst1b transcript lacking exon 6 in embryos injected with two different doses of the splice morpholino (MO2). (TIF) [file pone.0119040.s003.tif]

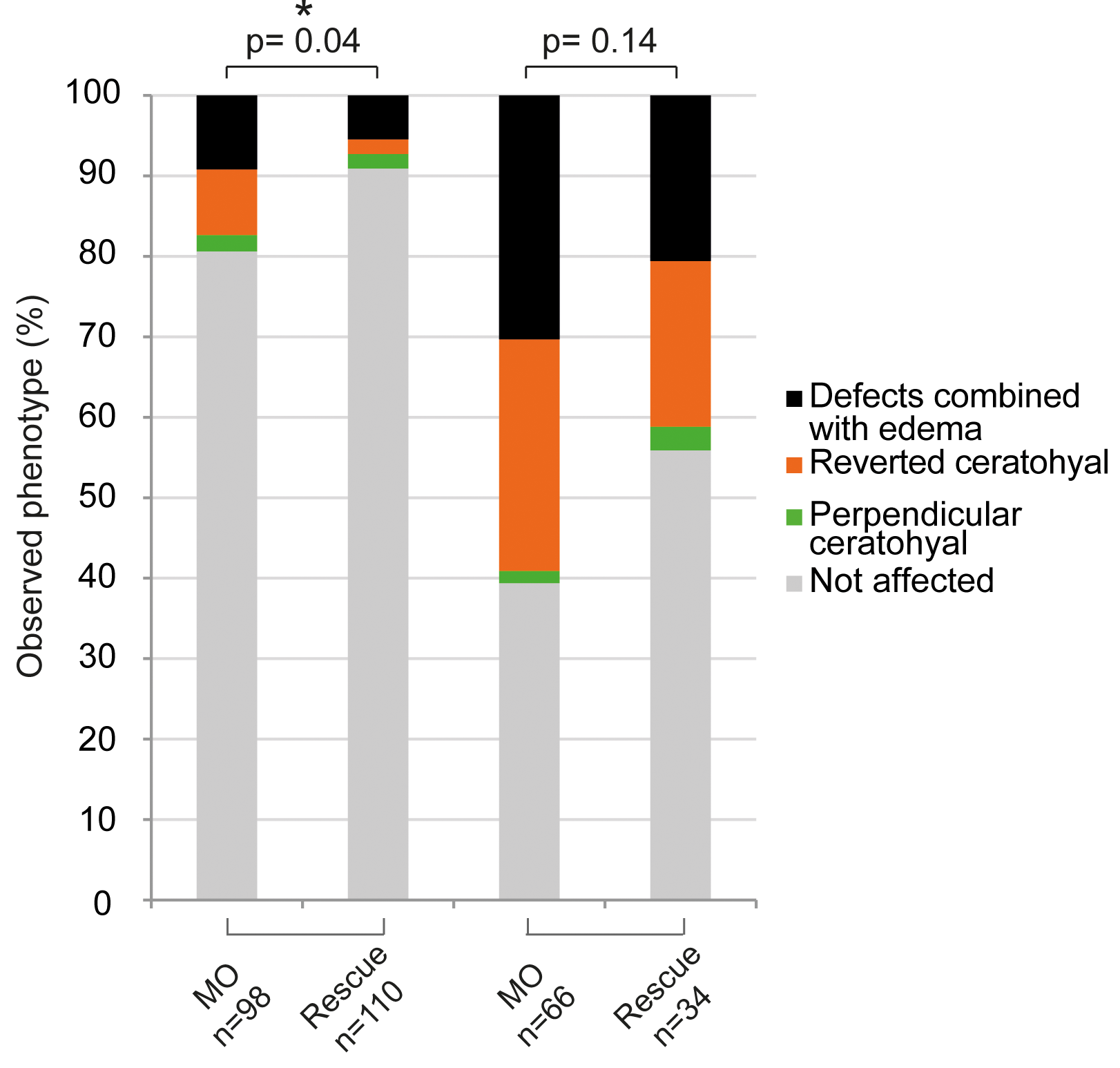

Supplement: S4 Fig — Embryos at 1–2 cell stages were injected with MO1 mixture and divided into two groups. Embryos in the “rescue” group were assigned to a second round of injection with Ndst1b mRNA. Both the proportion and severity of ceratohyal defects decreased when the larvae were injected with 22.5pg mouse Ndst1 mRNA. Larvae were classified as not affected (grey), or depending on increasing levels of defects in the second pharyngeal arch as perpendicular ceratohyal (green), reverted ceratohyal (orange) or those with cartilaginous defects combined with edema (black). For classification see Fig. 6. The phenotypic difference beteween embryos injected with morpolino only and those injected with morpholino and mouse Ndst1 mRNA is significant in the first experiment (* p<0.05, chi-square test, comparing affected and nonaffected embryos). (TIF) [file pone.0119040.s004.tif]

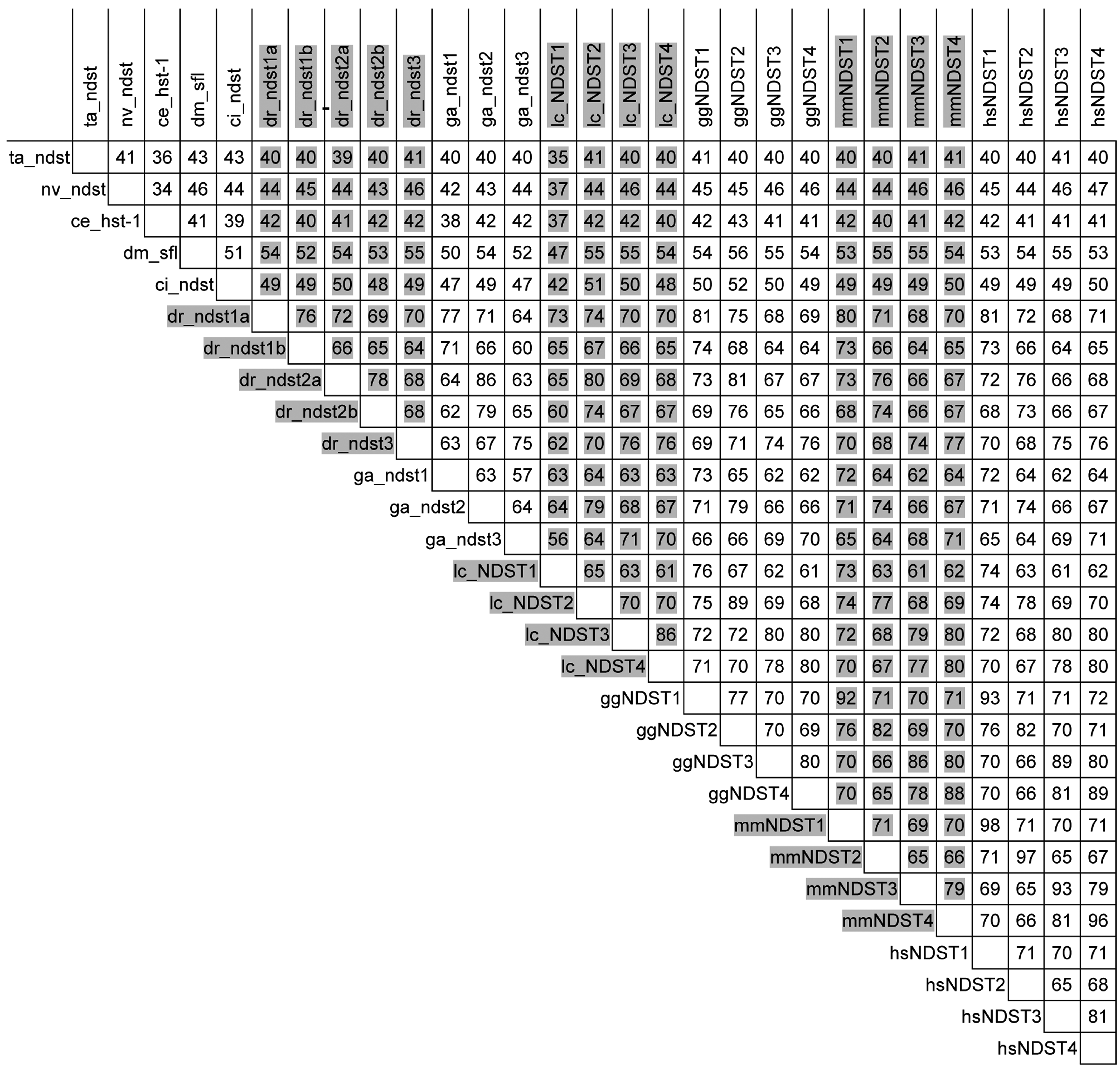

Supplement: S1 Table — Species names are abbreviated as follows: hs, human; mm, mouse; gg, chicken; lc, coelacanth; ga, stickleback; dr, zebrafish; ci, sea squirt; dm, fruit fly; ce, C. elegans; nv, starlet sea anemone; ta, Trichoplax. The accession numbers are listed under Experimental procedures. (TIF) [file pone.0119040.s005.tif]
